# Simultaneous Determination of Parathion, Malathion, Diazinon, and Pirimiphos Methyl in Dried Medicinal Plants Using Solid-Phase Microextraction Fibre Coated with Single-Walled Carbon Nanotubes

**DOI:** 10.1100/2012/627607

**Published:** 2012-05-02

**Authors:** Reza Ahmadkhaniha, Nasrin Samadi, Mona Salimi, Parisa Sarkhail, Noushin Rastkari

**Affiliations:** ^1^Pharmaceutical Sciences Research Center, Tehran University of Medical Sciences, Tehran 14174, Iran; ^2^Department of Drug and Food Control, Faculty of Pharmacy and Biotechnology Research Center, Tehran University of Medical Sciences, Tehran 14174, Iran; ^3^Physiology & Pharmacology Department, Pasteur Institute of Iran, Tehran 13164, Iran; ^4^Center for Air Pollution Research, Institute for Environmental Research, Tehran University of Medical Sciences, Tehran 14174, Iran

## Abstract

A reliable and sensitive headspace solid-phase microextraction gas chromatography-mass spectrometry method for simultaneous determination of different organophosphorus pesticides in dried medicinal plant samples is described. The analytes were extracted by single-walled carbon nanotubes as a new solid-phase microextraction adsorbent. The developed method showed good performance. For diazinon and pirimiphos methyl calibration, curves were linear (*r* ^2^ ≥ 0.993) over the concentration ranges from 1.5 to 300 ng g^−1^, and the limit of detection at signal-to-noise ratio of 3 was 0.3 ng g^−1^. For parathion and malathion, the linear range and limit of detection were 2.5–300 (*r* ^2^ ≥ 0.991) and 0.5 ng g^−1^, respectively. In addition, a comparative study between the single-walled carbon nanotubes and a commercial polydimethylsiloxane fibre for the determination of target analytes was carried out. Single-walled carbon nanotubes fibre showed higher extraction capacity, better thermal stability (over 350°C), and longer lifespan (over 250 times) than the commercial polydimethylsiloxane fibre. The developed method was successfully applied to determine target organophosphorus pesticides in real samples.

## 1. Introduction

Medicinal plants are widely consumed for pharmaceutical preparations and as a supplement for dietetic products and especially for “self-medication” in the general population, and they are also commonly used in health care products, food additives, or supplementary foods. Therefore, a large quantity of them is consumed in both daily life and pharmaceutical industries. In addition, herbal medicinal materials are increasingly favored by people because of their distinct curative effects and naturally physiologic properties. On the other hand, pesticides are often used in order to improve productivity and profit margins in the production of medicinal plants. Therefore, pesticide residues in herbal medicinal materials become pitfalls in safety and present obstacles to be acknowledged by the international community. Up to now, many international organizations and countries have set up regulations concerning the pesticides in the plants and plant products. In recent years, with the significant improvements in pesticide analysis and tremendous concerns in the safety of consumers' products, the pesticides residues in foods have been more strictly monitored in the aspects such as classes and/or amounts as well as MRLs (maximum residue level) [1]. The published researches on pesticide residues in crude herbal materials indicate that the presence of organophosphorus pesticide (OPP) residues is quite common [2–4].

Many different analytical procedures for the determination of pesticides in herbal materials have been reported [5–9]. The solid-phase microextraction (SPME) technique has found increasing use in recent years, as it is commercially available, rapid, simple, and inexpensive compared to other techniques [10, 11]. SPME integrates extraction, preconcentration, and cleanup in a single step, avoiding the use of organic solvents. Although commercial SPME fibres have been widely used, they still have some drawbacks such as low thermal-chemical stability and fragility of the support which restrict the application of these kind of fibres [12–14]. Therefore, it is of interest to develop a new low-cost SPME fibre with improved analytical properties, which can be easily prepared. We have developed a new SPME fibre using single-walled carbon nanotubes (SWCNTs) as adsorbent, which successfully applied for headspace solid-phase microextraction (HS-SPME) of target compounds in different matrices [15–18]. In the present paper, the feasibility of SWCNTs adsorbent for headspace SPME of OPPs in dried medicinal plant samples was investigated. By using SWCNT fibre, many of the analytical problems were solved, and considerable improvement was obtained. In addition, the superior performance of SWCNTs as material coating makes it possible to quantify all the target compounds simultaneously. To develop the HS-SPME method, SWCNTs were attached onto a stainless steel wire through an organic binder. Potential factors affecting the extraction efficiency were optimized, and the analytical performance of the developed SWCNT-coated fibre was compared with that of commercial PDMS fibre, which was frequently used for determination of OPPs in different matrices [1, 19–21]. The application of the developed method in OPPs residual analysis was shown by the simultaneous determination of trace amounts of parathion, malathion, diazinon, and pirimiphos methyl in some medicinal plant samples.

## 2. Experimental

### 2.1. Chemicals and Reagents

The SWCNTs synthesized by chemical vapor deposition process used as the material coating were 1-2 nm in diameter and 1–10 *μ*m in length (SWCNT-1, Nanoshel, Panchkula, India). The specific surface area and thermal conductivity of the SWCNTs were 350–450 m^2^ g^−1^ and  3000 ± 450 Wm^−1^ k^−1^, respectively. The pesticide standards parathion, malathion, diazinon, and pirimiphos methyl each with more than 98% purity and caffeine were obtained from Sigma-Aldrich (St. Louis, MO, USA). Standard stock solutions (1 mg mL^−1^) were prepared in HPLC-grade methanol and stored in the dark at 0°C. All other chemicals and solvents were of analytical-reagent grade or better.

### 2.2. Samples

Three samples each of *Zataria* (*Zataria multiflora*), *Chamomile* (*Matricaria recutita*), *Borage* (*Borago officinalis*), and *Spearmint* (*Mentha spicata*) were collected randomly from local markets in March 2010 in Tehran. The plant materials were powdered, sieved (1-2 mm), and stored away from light and moisture.

### 2.3. Instrumentation

SWCNT-coated fibres were prepared according to the previous study [15]. The average length and film thickness of the prepared SWCNT coating were determined as ~1 cm and ~92 *μ*m, respectively. Commercial 100 *μ*m PDMS fibres for comparative purpose and SPME holders were purchased from Supelco (Bellefonte, PA, USA). The instrument used for GC-MS analysis was an Agilent gas chromatograph 6890 plus (Agilent Technologies, Palo Alto, CA, USA) equipped with a 5973 quadrupole mass spectrometer. The gas chromatograph was fitted with an HP-5MS column (30 m, 0.25 mm i.d., 0.25 *μ*m film thickness). The instrumental temperatures were as follows: injector temperature, 230°C; initial oven temperature, 120°C (held for 3 min), increased to 260°C at a rate of 10°C min^−1^, held for 2 min, and then increased to the final temperature 300°C at a rate of 20°C min^−1^, where it was held for 1 min. The inlet was operated in splitless mode. The temperature of the transfer line was maintained at 300°C. Helium (99.999%) was used as carrier gas at 1.5 mL min^−1^ (constant flow). The source and quadrupole temperatures were kept at 230 and 150°C, respectively. The electronic beam energy of the mass spectrometer was set at 70 eV. The mass selective detector was operated in electron impact (EI) mode using selected ion monitoring (SIM). The dwell time of each ion was set at 100 ms. The GC conditions were selected to minimize the time of analysis while allowing all the analytes to elute in acquisition groups containing suitable number of ions for monitoring (Table 1).

### 2.4. Headspace Solid-Phase Microextraction

For the HS-SPME, manual SPME holders and different fibres were used. The fibres were conditioned at 20°C higher than the desorption temperatures. Two blank injections were performed before the actual analysis. Between uses, fibres were kept sealed from ambient air by piercing the tip of the SPME needle into a small piece of septum to prevent accidental contamination. The HS-SPME parameters were determined by experiments in which all the parameters were kept constant except one, and the remaining one was modified to find optimum conditions. For sample preparation, 1 g of each sample was transferred into a 25 mL HS-SPME glass vial containing 0.5 g of NaCl and 100 *μ*L of methanolic solution of caffeine (I.S., 1 *μ*g mL^−1^). Then 20 mL of water-methanol (90 : 10) solution was added to each sample, and each vial was sealed with a headspace aluminium cap and a teflon-faced septum. The mixture was mixed for 1 h with a glassy magnetic stirring bar at 25°C. Then the vials were immersed in a water bath (70 ± 0.5°C). Samples were let to equilibrate for 15 min before HS-SPME. Upon insertion of the SPME fibre into the vial, the fibre was exposed to the headspace over the sample for 35 min. During this step, the mixture was intensively stirred with a glassy magnetic stirring bar with constant velocity. The fibre was then retracted, removed from the vial, and placed immediately into the injector of the GC system. Injection was accomplished by extending the fibre in the heated inlet for 5 min, and the splitter was opened after 4 min. Preliminary studies indicated that the above procedure allowed for reproducible, quantitative transfer of target OPPs into the injector of the GC system. Blank samples, containing internal standard (I.S.) and quality control samples (QCs), were analyzed at the beginning, middle, and at the end of the sample queue. Each sample was extracted by the HS-SPME technique in triplicate, and the average response was considered for quantification. 

## 3. Results and Discussion

### 3.1. Characterization of SWCNT Fibre

The surface characteristics of the SWCNT and PDMS fibres were investigated by scanning electron micrograph (SEM) technique [15]. The coating of SWCNT fibre possesses a rough surface with considerable porosity, which results in larger surface area and higher extractive capacity than conventional polymeric phases. 

### 3.2. Optimization of HS-SPME Procedure for SWCNTs Fibre

**Figure 1:**
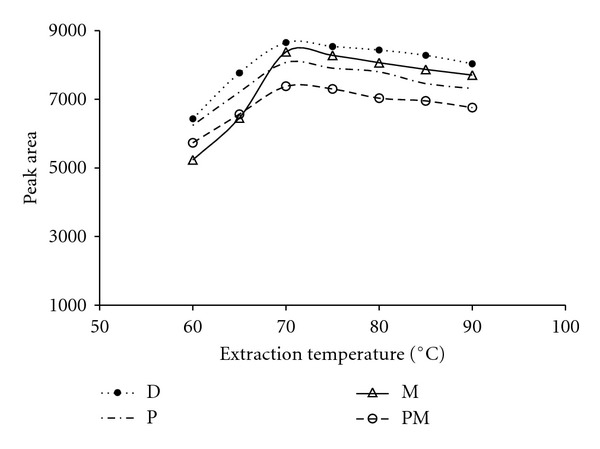
Extraction temperature profiles of SWCNT fibre for OPPs. Extraction time, 35 min; desorption time, 4 min; desorption temperature, 230°C; amount of NaCl, 25 mg mL^−1^. Abbreviations: malathion (M), parathion (P), diazinon (D), pirimiphos methyl (PM).

To develop a sensitive HS-SPME method, several experimental parameters related to both adsorption and desorption steps need to be optimized. Extraction temperature, an important parameter for the extraction process, may have two opposite effects on the extraction efficiency: high temperature can increase the distribution of analytes in gas phase while at the same time decrease the adsorption on the extraction medium. So optimizing the extraction temperature is necessary. The effect of temperature on the extraction efficiency was studied from 30 to 90°C. The results are demonstrated in Figure 1. Considerable decreases in sensitivity for target analytes were observed at temperature above 70°C, which could be the overall result of different effects [14, 18]. Based on the results, 70°C was chosen as extraction temperature for further studies. 

Salting-out effect has been well established in the previous works through adding different salts (mostly NaCl and Na_2_SO_4_) to the samples. Most authors agree on the positive effect of salt addition to the sample for improving the extraction efficiency [20, 22]. In this study, to investigate the effect of salt addition on the extraction efficiency, a series of experiments were performed by adding different amounts of NaCl and Na_2_SO_4_ (from 0 to 2 g) into the quality control samples (20 mL). According to the results, no significant differences were found between the salts, and the highest extraction efficiencies were obtained by adding 0.5 g of NaCl into the sample. Therefore, 0.5 g of NaCl per 20 mL of sample was selected as the optimum condition in the following experiments. 

**Figure 2:**
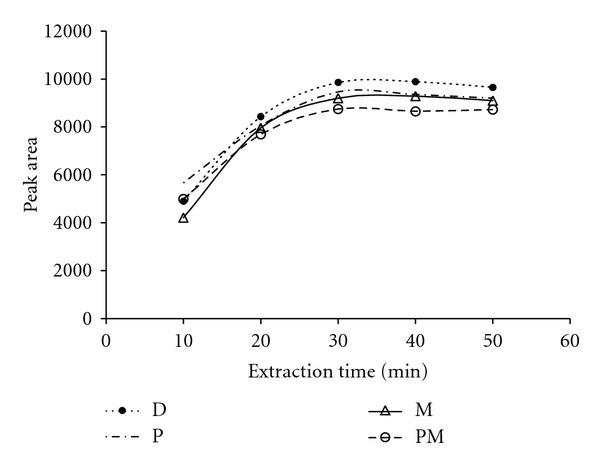
Extraction time profiles of SWCNT fibre for OPPs. Extraction temperature, 70°C; desorption time, 4 min; desorption temperature, 230°C; amount of NaCl, 25 mg mL^−1^. Abbreviations: malathion (M), parathion (P), diazinon (D), pirimiphos methyl (PM).

Extraction time has a significant effect on extraction yield. The effect of extraction time was investigated in the range 10–50 min (Figure 2). 

 The extraction amounts of target compounds increased with increasing exposure time, up to 30 min, and remain at steady state until the end of experiment (50 min). Considering these results, 35 min was adopted as extraction time in the following experiments. 

For the desorption process, two parameters, including desorption temperature and time, need to be optimized. The temperature used for desorption ranged from 100 to 290°C. According to the results, the peak areas of the analytes increased with increasing the desorption temperature. Since the best peak shapes were obtained at 230°C, this temperature was selected as the desorption temperature. Experiments showed that no carry-over effect took place at 230°C. 

Experiments with different desorption times from 0.5 to 6 min were carried out, and the results showed that desorption was completed in 4 min. Therefore, a 4 min desorption time and 230°C desorption temperature were used as optimum condition in all experiments. 

### 3.3. Optimization of HS-SPME Procedure for PDMS Fibre

The same methodology as the one described above was used to find the optimum HS-SPME condition for PDMS fibre, and the optimum condition was determined as follows: extraction time, 30 min; extraction temperature, 60°C; desorption time, 5 min; desorption temperature, 240°C; concentration of NaCl, 50 mg mL^−1^. 

### 3.4. Comparing SWCNT Fibre with PDMS Fibre

**Figure 3:**
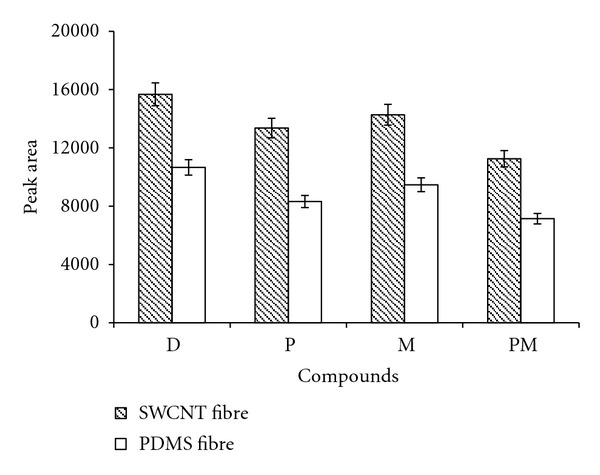
Comparison of extraction efficiency between SWCNT fibre and PDMS fibre (target analyte, diazinon: 75 ng g^−1^). Experimental conditions for SWCNT fibre: extraction time, 35 min; extraction temperature, 70°C; desorption time, 4 min; desorption temperature, 230°C; amount of NaCl, 25 mg mL^−1^ and for PDMS fibre: extraction time, 30 min; extraction temperature, 60°C; desorption time, 5 min; desorption temperature, 240°C; amount of NaCl, 50 mg mL^−1^. Abbreviations: malathion (M), parathion (P), diazinon (D), pirimiphos methyl (PM).

The choice of the most suitable coating is very important for achieving good selectivity and sensitivity. Commercial PDMS fibre, which was frequently used for determination of OPPs [1, 19–21], is selected for comparing with SWCNT fibre developed in the previous studies [15–18]. Comparison of the extraction yields of commercial PDMS and SWCNT fibre for 75 ng g^−1^ OPPs was carried out under the optimal HS-SPME condition of each fibre, and the results are represented in Figure 3. These results indicate the higher extraction efficiency of SWCNT coating, which is due to the high adsorption ability of SWCNT fibre. 

In HS-SPME, thermal stability and lifespan are the crucial properties of fibre coating. Generally, for commercial PDMS fibre, the thermal stability is lower than 250°C, and the range of lifetime is from 50 to 100 times. The thermal stability of SWCNT fibre was studied, and it was found that the extraction efficiency of SWCNT fibre is not significantly affected after the fibre was conditioned for 1 h at 250, 300, and 350°C. These results indicate the considerable thermal stability of SWCNT fibre. The change of extraction efficiency of SWCNT fibre in extracting OPPs from the aqueous solution after being used for more than 100 times was also studied. The results indicated that the extraction efficiency of SWCNT fibre has no obvious decline after being used for 250 times. On the other hand, most of the commercial fibres are breakable due to the use of fused silica as substrate for coating. The developed SWCNT fibre was unbreakable since stainless steel wire was used as the core. These advantages of SWCNT fibre expand the HS-SPME-GC application range toward higher-boiling compounds and prolong the fibre lifetime. 

### 3.5. Quantitative Analysis

The calibration curves parameters listed in Table 2 were obtained under the optimized condition. 

Linearity of the calibration curves was determined in the ranges 1.5–300 ng g^−1^ (for diazinon and pirimiphos methyl) and 2.5–300 ng g^−1^ (for malathion and parathion). Coefficient of estimation ranged from 0.991 to 0.993. The LOD was defined as three times the standard deviation of baseline noise (*n* = 6) and was determined by spiking serially diluted analyte standards into blank samples. According to the ICH (International Conference on Harmonization of Technical Requirements for Analytical Methods) guideline for analytical method validation, limit of quantification (LOQ) for each analyte was determined as the lowest concentration on the calibration curve with a precision of less than 20% coefficient of variation (CV%) and an accuracy of 80–120% [23]. For diazinon and pirimiphos methyl, LOD and LOQ were determined as 0.3 and 1.5 ng g^−1^, respectively, and for malathion and parathion, LOD and LOQ were determined as 0.5 and 2.5 ng g^−1^, respectively, which are better or comparable with that of reported methods [1, 3, 6, 8–10]. The precision of the method was evaluated in terms of intermediate precision (or interday precision) through calculating the analyte concentration in quality control samples, prepared at three levels (each six replicates) on three consecutive days. Interday precision values for the analytes were always less than 14% (Table 3). Expression of the repeatability (or intraday precision) is based on the CVs of determined responses of six replicates of quality control (QC) samples, which were prepared at three levels and reported in Table 3. The estimated recoveries at three different concentration levels are also shown in Table 3. To determine the recovery, mean peak area of each analyte at each concentration level was determined for a blank sample spiked with the analyte and compared with that of standard solution at the same concentration. Fibre reproducibility was evaluated with QC samples (75 ng g^−1^) through headspace extraction. Batch-produced five fibres were used for the evaluation of the reproducibility between fibres. 

**Figure 4:**
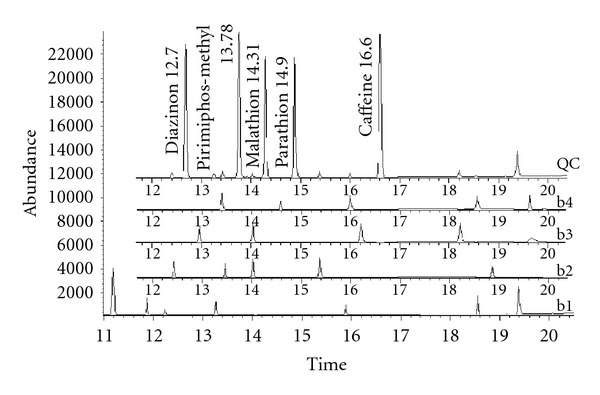
Representative SIM GC-MS chromatograms; (QC) a quality control sample (a mixture of blank samples spiked with the analytes at 75 ng g^−1^), (b1–b4) blank samples; (b1) *Zataria*, (b2) *Chamomile*, (b3) *Borage*, and (b4) *Spearmint*.

As shown in Table 3, the reproducibility between the SWCNT fibres for headspace extraction of the OPPs was acceptable (10.8% < RSD < 12.4%). The results proved the feasibility of the fibre preparation method. All these results indicate the feasibility and reliability of the developed method for determining parathion, malathion, diazinon, and pirimiphos methyl in dried plant samples. The selectivity of the method was confirmed by analyzing three different samples of each target plant which have not been treated with OPPs (wild species). There was no interfering peak in the region of the analytes and internal standard (Figure 4). 

### 3.6. Determination of Parathion, Malathion, Diazinon, and Pirimiphos Methyl in Real Samples

**Figure 5:**
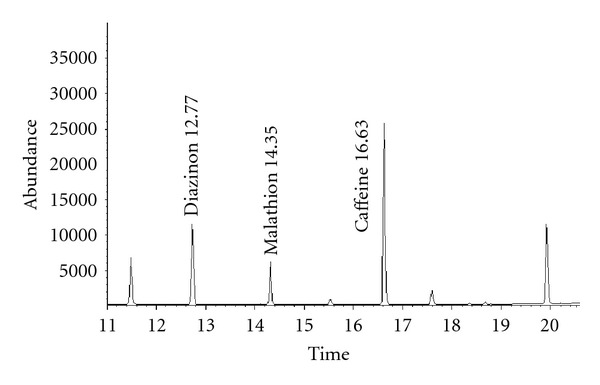
Representative SIM GC-MS chromatogram of a Chamomile sample. Quantification results: diazinon (53.1 ng g^−1^) and malathion (22.9 ng g^−1^).

Parathion, malathion, diazinon, and pirimiphos methyl were analyzed simultaneously in twelve medicinal plants samples. Determined concentrations of the OPPs are listed in Table 4. A chromatogram of one of *Chamomile* samples is shown in Figure 5. 

 A significant difference between different plant samples and also between OPPs was observed. Furthermore, the mean concentrations of malathion, diazinon in *Zataria*, and *Chamomile* were significantly greater than those of other medicinal plants (*ANOVA, P* < 0.05). These results are in accordance with others who found similar levels of OPPs in other medicinal plant samples [8, 9, 24]. The results of this study indicate applicability of the developed method for monitoring OPPs in medicinal plant samples. 

## 4. Conclusion

 The performance of the SWCNT-coated fibre for simultaneous determination of some OPPs in plant samples was evaluated and based on the results, the SWCNT fibre showed a higher sensitivity and longer lifespan (over 250 times) than the commercial PDMS fibre, as well as good precision and high thermal stability. The other advantages of the developed SPME fibre in comparison with the commercial fibres are the ease of preparation, physical resistance to damage, and low cost. By using SWCNTs fibre, a simple, specific, and sensitive HS-SPME GC-MS method for the determination of OPPs in medicinal plants with the total analysis (sample preparation and instrumental analysis) time being 72 min has been developed and validated. The optimum HS-SPME condition for SWCNTs fibre was determined as extraction time, 35 min; extraction temperature, 70°C; desorption time, 4 min; desorption temperature, 230°C; concentration of NaCl, 25 mg mL^−1^. The LODs for the analytes were determined in the range of 0.3 to 0.5 ng g^−1^ which is better or comparable with that of reported methods. By using the developed method, the mean concentrations of target analytes were determined in the analyzed samples as parathion (<0.5 ng g^−1^), diazinon (4.2–130.2 ng g^−1^), malathion (2.7–25.1 ng g^−1^), and pirimiphos methyl (<0.3 ng g^−1^). By considering the analytical features of the developed method, it may also be applicable to other kinds of dried plant samples such as vegetables and fruits. 

## Figures and Tables

**Table 1:** Selected ions used for the quantification and qualification of target analytes by GC-MS (SIM mode).

Ion group	Analyte	Time window (min)	Molecular ion (*m/z*)	Quantification ion (*m/z*)	Confirmation ions (*m/z*)
1	Diazinon	12-13	304	179	276, 304
2	Pirimiphos methyl	13-14	305	290	276, 305
3	Malathion	14–16	330	173	158, 256
3	Parathion	14–16	291	291	263, 235
4	Caffeine	16-17	194	194	109, 165

**Table 2:** Calibration curve parameters of the developed HS-SPME method for determination of OPPs in medicinal plant samples.

Target compound	Linear range (ng g^−1^)	Limit of detection (LOD) (ng g^−1^)	Calibration curve equation	Coefficient of estimation (*r* ^2^)
Diazinon	1.5–300	0.3	*y* = 0.97*x* + 0.214	0.993
Pirimiphos methyl	1.5–300	0.3	*y* = 0.91*x* − 0.014	0.993
Malathion	2.5–300	0.5	*y* = 0.86*x* + 0.087	0.991
Parathion	2.5–300	0.5	*y* = 0.83*x* − 0.127	0.991

**Table 3:** Estimated recoveries, accuracies, and precisions for determination of the analytes at different concentrations (*n* = 6) in QC samples.

Target compound	sample	Nominal concentration (ng g^−1^)	Mean of calculated concentration (ng g^−1^)	CV(%) of calculated concentration (interday)	CV(%) of calculated concentration (intraday)	RE(%) of calculated concentration^a^	Estimated recoveries (%)	CV(%) of calculated recovery	RSD between fibres (%)	
									SWCNTS	PDMS
Diazinon	QC_1_	3	2.6	11.8	11.2	−13.3	78	12.1		
	QC_2_	75	70.4	10.3	10.1	−6.1	82	10.2	10.8	6.7
	QC_3_	275	261	9.5	8.2	−5.1	87	8.8		
Pirimiphos methyl	QC_1_	3	2.7	11.5	11.2	−10.0	77	11.7		
	QC_2_	75	69.7	10.7	10.4	−7.1	84	10.5	11.6	7.8
	QC_3_	275	258	9.7	9.5	−6.2	85	8.7		
Malathion	QC_1_	3	3.4	13.1	12.7	13.3	75	12.7		
	QC_2_	75	78.4	12.2	11.8	4.5	79	11.8	11.2	6.5
	QC_3_	275	285.7	10.7	9.8	3.9	82	10.6		
Parathion	QC_1_	3	2.7	12.6	12.2	−10.0	81	12.4		
	QC_2_	75	70.3	11.6	11.3	−6.3	84	11.3	12.4	6.3
	QC_3_	275	266.7	10.4	9.8	−3.0	83	9.8		

Note:  ^*a*^Relative error = [(calculated concentration/nominal concentration)−1]×100.

**Table 4:** Levels of malathion, diazinon, parathion, and pirimiphos methyl in studied medicinal plant samples (ng g^−1^).

Groups	No.	Diazinon	Malathion	Parathion	Pirimiphos methyl
*Borage*	1	7.44	n.d	n.d.	n.d.
	2	n.d	3.35	n.d.	n.d.
	3	5.64	n.d	n.d.	n.d.
*Zataria *	1	98.55	20.43	n.d.	n.d.
	2	64.54	25.15	n.d.	n.d.
	3	130.25	14.34	n.d.	n.d.
*Chamomile*	1	53.15	22.93	n.d.	n.d.
	2	43.24	15.15	n.d.	n.d.
	3	49.65	10.54	n.d.	n.d.
*Spearmint*	1	12.54	6.53	n.d.	n.d.
	2	10.15	2.74	n.d.	n.d.
	3	4.25	4.83	n.d.	n.d.

Note: n.d.: not detected.
